# Selective blockade of B7‐H3 enhances antitumour immune activity by reducing immature myeloid cells in head and neck squamous cell carcinoma

**DOI:** 10.1111/jcmm.13143

**Published:** 2017-04-11

**Authors:** Liang Mao, Teng‐Fei Fan, Lei Wu, Guang‐Tao Yu, Wei‐Wei Deng, Lei Chen, Lin‐Lin Bu, Si‐Rui Ma, Bing Liu, Yansong Bian, Ashok B. Kulkarni, Wen‐Feng Zhang, Zhi‐Jun Sun

**Affiliations:** ^1^ The State Key Laboratory Breeding Base of Basic Science of Stomatology & Key Laboratory of Oral Biomedicine Ministry of Education Wuhan University Wuhan China; ^2^ Department of Oral Maxillofacial‐Head Neck Oncology School and Hospital of Stomatology Wuhan University Wuhan China; ^3^ Gastrointestinal Oncology Section Thoracic and Gastrointestinal Oncology Branch National Cancer Institute National Institutes of Health Bethesda MD USA; ^4^ Functional Genomics Section Laboratory of Cell and Developmental Biology National Institute of Dental and Craniofacial Research National Institutes of Health Bethesda MD USA

**Keywords:** B7‐H3, HNSCC, immunotherapy, myeloid‐derived suppressor cells, tumour‐associated macrophages

## Abstract

Immature myeloid cells including myeloid‐derived suppressor cells (MDSCs) and tumour‐associated macrophages (TAMs) promote tumour growth and metastasis by facilitating tumour transformation and angiogenesis, as well as by suppressing antitumour effector immune responses. Therefore, strategies designed to reduce MDSCs and TAMs accumulation and their activities are potentially valuable therapeutic goals. In this study, we show that negative immune checkpoint molecule B7‐H3 is significantly overexpressed in human head and neck squamous cell carcinoma (HNSCC) specimen as compared with normal oral mucosa. Using immunocompetent transgenic HNSCC models, we observed that targeting inhibition of B7‐H3 reduced tumour size. Flow cytometry analysis revealed that targeting inhibition of B7‐H3 increases antitumour immune response by decreasing immunosuppressive cells and promoting cytotoxic T cell activation in both tumour microenvironment and macroenvironment. Our study provides direct *in vivo* evidence for a rationale for B7‐H3 blockade as a future therapeutic strategy to treat patients with HNSCC.

## Introduction

HNSCC is the seventh most common cancer worldwide, with more than 600,000 new cases reported each year 1. Despite advances in diverse therapeutic approaches, the survival rate of patients with HNSCC has shown minimal improvement, and this persistent high mortality is largely due to high rates of regional metastasis, locoregional recurrence and drug resistance to current chemotherapy regimens 2, 3. Although many factors contribute to treatment failure in HNSCC, profound immune defects were consistently found in these patients. These defects include generalized T cell anergy and increased levels of MDSCs and TAMs 4, 5.

Since the mid‐1990s, it has been shown that MDSC recruitment at the tumour site is associated with an increased rate of metastasis and recurrence 6. MDSC subsets can be identified by the surface expression of CD11b and Gr‐1 in mice 7. In humans, MDSCs were first identified in the peripheral blood of patients with HNSCC. MDSC‐based T cell immune suppression at the tumour site is non‐antigen‐specific, and therefore, MDSCs also share the mechanisms by which they abrogate T cell proliferation and functionality, such as arginine metabolism by NOS2 and arginase‐1 or the production of ROS 7. TAMs are regarded as M2‐type macrophages, which are alternatively activated in solid tumour 8. TAMs promote all phases of tumorigenesis, such as tumour growth, invasion and metastasis, as well as stimulating tumour‐promoting processes such as angiogenesis and immune suppression 7, 9. Elevated expression of a number of monocyte chemoattractants, including chemokine (C‐C motif) ligand 2 (CCL2), CCL3, CCL4, CCL8 and CCL5 (RANTES), by both tumour and stromal cells within tumours has been shown to positively correlate with increased number of MDSCs and TAMs in many human tumours 10, 11, 12, 13, 14, 15.

Recent reports indicate that immune checkpoint blockade antibodies (e.g. anti‐CTLA‐4 and anti‐PD‐1) enhance T cell antitumour activity and have produced exciting and durable results in treatment of a number of cancers 16. B7 homolog 3 protein (B7‐H3) is a newly identified member of the B7 family. B7‐H3 is a transmembrane protein with immunoglobulin‐like structure 17, which is known to have immunoregulatory properties with both inhibitory and stimulatory effects on the activation of T cells 18, 19. However, the relationship between the B7‐H3 expression and clinical features in patients with HNSCC remains unclear, and preclinical evidence revealing the antitumour immune response of B7‐H3 especially in immature myeloid cells is still uncertain.

In this study, we demonstrated that a significant increase in B7‐H3 expression is an important immunosuppressive mechanism in human and mouse HNSCC. Oncogene activation by the conditional knockout of *Pten* and *Tgfbr1* may contribute to the tumour‐immunosuppressive microenvironment with concomitantly significant increase in MDSCs and TAMs. Moreover, we found that the blockade of B7‐H3 significantly reduces MDSCs and TAMs, as well as promoting IFN‐γ secretion of cytotoxic T cells in our HNSCC mouse model. These findings indicate that targeting B7‐H3 may be a potentially effective therapeutic approach to treat patients with HNSCC.

## Materials and methods

### *Tgfbr1/Pten* 2cKO mice

The time‐inducible tissue‐specific *Tgfbr1/Pten* 2cKO mice (*Tgfbr1* ^*flox/flox*^ *; Pten* ^*flox/flox*^ *; K14‐CreER* ^*tam+/−*^, FVBN/CD1/129/C57 mixed background) were maintained, genotyped and induced with oral application of tamoxifen (2 mg/kg in 200 μl corn oil by five consequent days) as previously described 20. *Tgfbr1* ^*flox/flox*^ *; Pten* ^*flox/flox*^ *; K14‐CreER* ^*tam−/−*^ mice from the same cage with same dose tamoxifen were used as wild‐type control 20. Mice were housed in the pathogen‐free Experimental Animal Center of Wuhan University in pressurized ventilated cages according to institutional regulations. All proposals were approved and supervised by the Institutional Animal Care and Use Committee of the Wuhan University.

### Animal experimental protocol

The antimouse B7‐H3‐blocking *in vivo* monoclonal antibody (MJ18, rat IgG1) purchased from BioXcell (West Lebanon, NH, USA) was stored at 4°C in a concentration of 6.96 mg/ml. The working solution was further diluted in PBS with a final concentration of 1 mg/ml immediately before use. The *in vivo* isotype control (clone: HRPN, rat IgG1) was used for prophylactic tumorigenesis experiments. After oral gavage with tamoxifen (2 mg/kg) for five consequent days (day 1 to day 5), the *Tgfbr1/Pten* 2cKO mice were intraperitoneally injected with 0.3 mg of MJ18 every other day starting from day 14 (0.3 mg/mouse, MJ18, i.p.; *n* = 5 mice). Isotype control mice received isotype IgG1 (0.3 mg/mouse, HRPN, i.p.; *n* = 5 mice). All animals were routinely inspected and monitored every other day. Tumour size was measured with a micrometer calliper and photographed every other day. The end‐point was determined according to a systematic evaluation by the veterinary doctor. The mice were killed at the end of the study, the immune organ and the tumour were harvested as soon as possible, and tissues were fixed in paraffin overnight or frozen at ‐80°C for immunostaining or Western blot analysis.

### Flow cytometry analysis

The single‐cell suspension was obtained from the spleen, lymphocyte node (LN), blood and tumour of HNSCC mouse model as previously described 21. The following antimouse antibodies were used for staining: FITC‐conjugated CD4, CD8 and CD11b, PE‐conjugated B7‐H3 and Gr‐1 (all from Becton Dickinson, Mountain View, CA, USA); PerCP‐Cy5.5‐conjugated F4/80, PE‐conjugated IFN‐γ, mouse regulatory T cell staining kit #3 (all from eBioscience, San Diego, CA, USA); and isotype‐matched IgG controls (eBioscience). The cells were analysed using FlowJo (Tree Star, Ashland, OR, USA) and gated by the side scatter and forward scatter filters. Dead cells were excluded by staining 7AAD (Invitrogen, Carlsbad, USA).

### Western blot

Spontaneous tumours that developed in *Tgfbr1/Pten* 2cKO mice were lysed in a T‐PER buffer containing 1% phosphatase inhibitors and complete mini cocktail (Roche, Basle, Switzerland). Detailed procedures of immunoblotting were described previously 20. In brief, proteins from each sample were denatured and then loaded in each lane of NuPAGE 4‐12% Bis‐Tris precast gel. Subsequently, proteins were transferred onto a NC membrane and blocked with 5% non‐fat milk for 1 hour, and then incubated with primary antibodies overnight and finally with horseradish peroxidase‐conjugated secondary antibody (Pierce, Rockford, IL, USA). The following primary antibody dilutions were used: 1:1000 for B7‐H3, p‐STAT3^T705^, CXCL1, CCL2 and GAPDH.

### Human HNSCC tissue array

On approval from the School and Hospital of Stomatology of Wuhan University Medical Ethics Committee, the informed consents were obtained from the patients. Further details have previously been described 21. Custom‐made tissue arrays were used for immunohistochemistry staining. The tissue microarray slides included 165 confirmed cases of HNSCC, 48 cases of normal oral mucosa and 45 cases of oral epithelial dysplasia (Outdo Biotech, Shanghai, China) 22. Disease status of the patients in the HNSCC tumour registry is updated each year, and patient vital status is updated on a yearly basis.

### Immunohistochemistry

The tissue array sections were stained as per the previous protocol 22. In brief, the sections were incubated overnight at 4°C with antibody for B7‐H3 (Cell Signaling Technology, Danvers, MA, USA), CD8 (Zymed, Shanghai, China), CD68 (Zymed), CD163 (CWBiotech, Beijing, China), CD11b (Abcam, Cambridge, UK), CD33 (Zymed), p16 (Zymed), granzyme B (Zymed) or isotype‐matched IgG controls. Then, the sections were incubated with a secondary biotinylated immunoglobulin G antibody solution and an avidin–biotin–peroxidase reagent. At last, after being washed three times with phosphate‐buffered saline, the sections were lightly counterstained with Mayer's haematoxylin (Invitrogen). Slides were scanned by an Aperio ScanScope CS scanner (Aperio Technologies, Vista, CA, USA). The scanned image data were then processed with background subtraction and white balance. IHC staining of the membrane, nuclei or pixel was quantified by quantitative software Aperio version 12.1. Histoscore was defined to describe the quantification of staining as below. Scanned high‐power field of each sample, membrane and nuclear immunostaining was calculated by the formula: (1 × the percentage of weakly positive staining) + (2 × the percentage of moderately positive staining) + (3 × the percentage of strongly positive staining). Histoscore of pixel quantification was calculated as total intensity/total cell number. The threshold for scanning of different positive cells was set by the standard controls provided by Aperio.

### Immunofluorescence

Tumour sections were hydrated in alcohol, washed three times in PBS, retrieved using sodium citrate in a pressure cooker, blocked with 2.5% bovine serum album in PBS buffer for 1 hour at 37°C and then incubated with primary antibody or isotype‐matched IgG controls overnight at 4°C. The next day, slides were incubated with fluorochrome‐conjugated secondary antibodies (Alexa 594 anti‐rabbit and Alexa antimouse; Invitrogen) and mounted in Vectashield with 4′,6‐diamidino‐2‐phenylindole (DAPI; Vector Laboratories, Burlingame, USA). Fluorescence images were then captured using a Zeiss CLSM‐310 fluorescence microscope.

### Hierarchical clustering, data visualization and statistical analysis

As reported previously, hierarchical clustering was performed using Cluster program with average linkage based on Pearson's correlation, visualized using the TreeView program. Data analyses were carried out using GraphPad Prism version 5.0 for Windows (GraphPad Software Inc., La Jolla, CA, USA). One‐way anova followed by the post hoc Tukey multiple comparison tests and unpaired *t*‐test was used to analyse the differences in positive cells and immunostaining within each group. Two‐tailed Pearson's correlation statistic was used for correlation between B7‐H3, CD11b, CD33, CD68 and CD163. Kaplan–Meier analysis followed by log‐rank test was used to compare the survival of patients in the two groups. Mean values ± S.E.M. with a difference of *P* < 0.05 were considered statistically significant (ns, *P* > 0.05, *P < 0.05; **P < 0.01; ***P < 0.001).

## Results

### Overexpression of B7‐H3 in human HNSCC confers poor prognosis of patients with HNSCC

**Figure 1:**
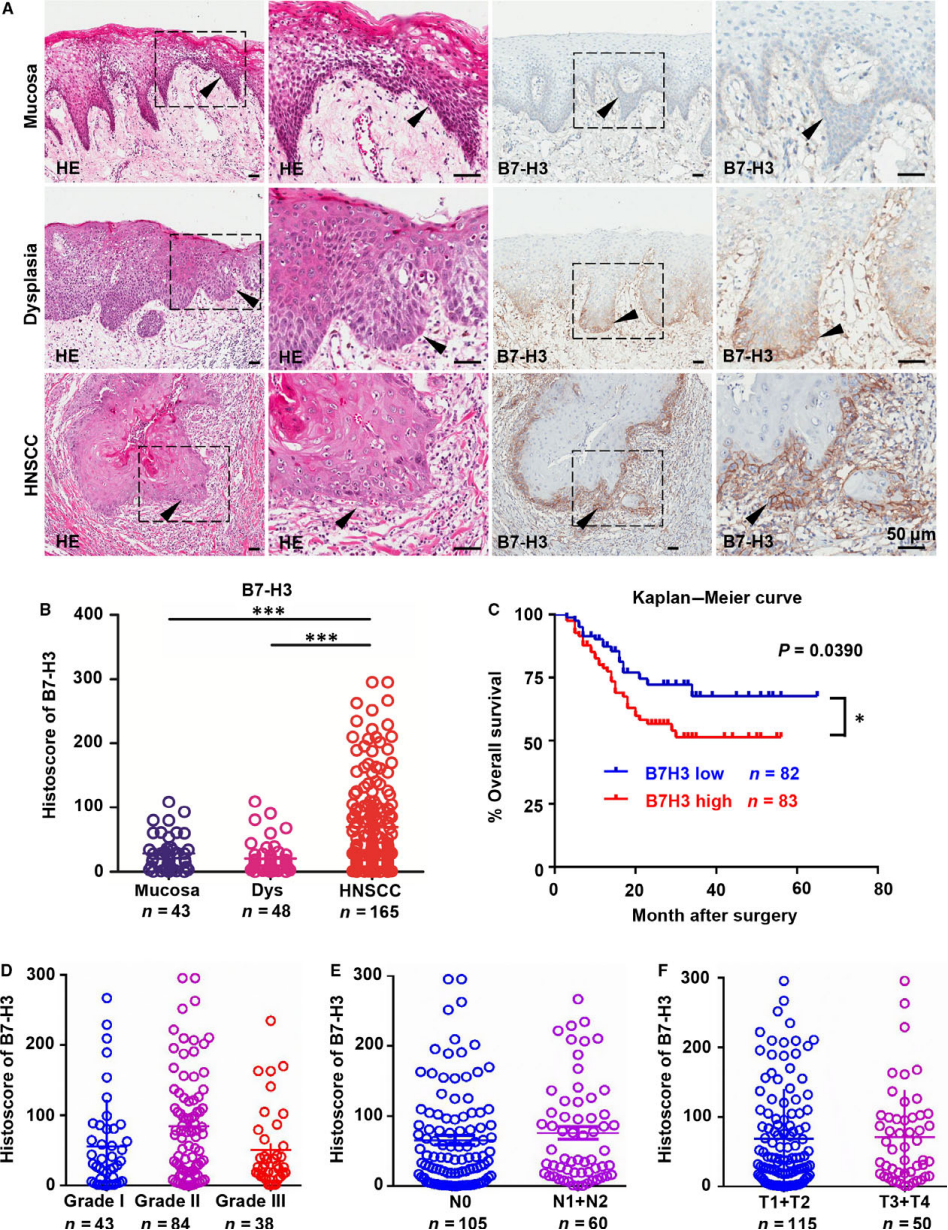
Overexpression of B7‐H3 in human HNSCC confers poor prognosis of patients with HNSCC. Representative haematoxylin–eosin, immunohistochemical staining (arrowhead) (**A**) and quantification (**B**) of B7‐H3 in human head and neck squamous cell carcinoma (HNSCC,* n* = 165) as compared with dysplasia (Dys, *n* = 48) and oral mucosa (*n* = 43). Scale bar, 50 μm (GraphPad Prism 5, one‐way ANOVA followed by post hoc Tukey; each dot is presented as an independent core which indicates the IHC staining, ***P < 0.001). (**C**) Kaplan–Meier survival analysis indicated that high B7‐H3 expression confers poor overall survival in patients with HNSCC (*n* = 165, *P < 0.05, *P*‐value =0.0390) with median expression of B7‐H3 as a cut‐off (GraphPad Prism 5, log‐rank test). B7‐H3 staining was not significantly alternated in different pathological grades (**D**), lymph node metastasis status (**E**) and tumour size (**F**) (GraphPad Prism 5, one‐way ANOVA followed by post hoc Tukey; each dot is presented as an independent core).

A recent study suggests that B7‐H3 may overexpress in human patients with HNSCC 23. To identify mRNA and DNA expression in human HNSCC, we searched the publicly available cancer data set in the Oncomine database 24. CD276 (encoding B7‐H3) mRNA or DNA copy number was significantly increased in eight of 17 data sets (cut‐off: *P* < 0.05, fold change >1.5). In a meta‐analysis consisting eight data sets of head and neck cancer gene expression profiling, the mRNA level of CD276 was significantly increased in HNSCC compared with the control counterpart (*P* < 0.05; Fig. S1). Based on this finding, we quantified the expression of B7‐H3 in human HNSCC by immunohistochemistry. The staining included 48 cases of oral mucosa, 43 cases of dysplasia (Dys) and 165 cases of HNSCC specimens (Fig. 1A and B). As shown in Fig. 1A, positive immunostaining of B7‐H3 was mainly found in cancer cells, especially in the invasive frontier of cancer. Also, immunoreactivity of B7‐H3 can be detected in stromal and infiltrating inflammatory cells (Fig. 1A). Immunostaining of B7‐H3 was significantly increased in human HNSCC tissue as compared with rather lower expressions in oral mucosa (*P* < 0.001) and dysplasia (*P* < 0.001). We further analysed the clinicopathological characteristics of HNSCC. Of interest, Kaplan–Meier survival analysis indicated that high expression of B7‐H3 confers poor overall survival in patients with HNSCC (*n* = 165, *P* = 0.0390; Fig. 1C) with median expression of B7‐H3 as a cut‐off. Particularly, we found that B7‐H3 staining was not significantly altered in different pathological grades, lymph node metastasis status and tumour size (Fig. 1D‐F).

### Overexpression of B7‐H3 in human HNSCC is associated with immature myeloid cells

**Figure 2:**
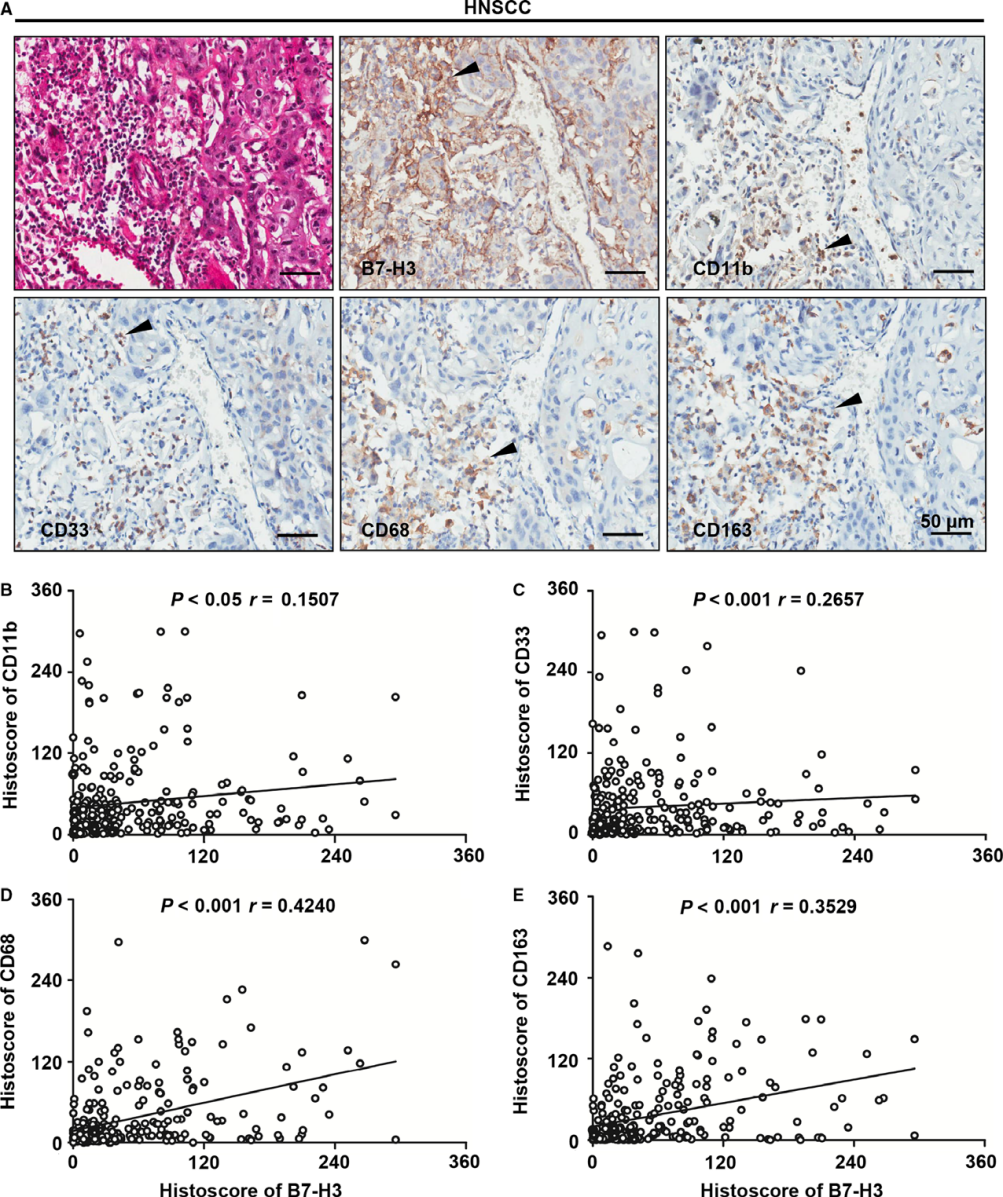
Overexpression of B7‐H3 in human HNSCC associated with immature myeloid cell. (**A**) Representative immunohistochemical staining of B7‐H3, CD11b, CD33, CD68 and CD163 in human HNSCC specimens. Scale bar, 50 μm. The expression of B7‐H3 was positively correlated with CD11b (*P* < 0.05, *r* = 0.1507) (**B**), CD33 (*P* < 0.001, *r* = 0.2657) (**C**), CD68 (*P* < 0.001, *r* = 0.4240) (**D**) and CD163 (*P* < 0.001, *r* = 0.3529) (**E**) in human HNSCC (statistics including 165 primary HNSCC; GraphPad Prism 5, two‐tailed Pearson's correlation).

Cumulative studies demonstrated that suppressive myeloid cells, including MDSCs and TAMs, play an indispensable role in tumorigenesis and tumour progression. This finding prompted us to detect the correlation between the B7‐H3 and suppressive myeloid cells. We analysed CD11b, CD33, CD68 and CD163 by immunohistochemical staining in human HNSCC as shown in Fig. 2A. The expression of B7‐H3 was positively correlated with CD11b and CD33 (markers for human MDSCs) (Fig. 2B). Similarly, the expression of CD68 and CD163 (markers for M2‐type macrophages) was positively correlated with the B7‐H3 (Fig. 2A‐C). Together, these results suggest that the activation of B7‐H3 is strongly correlated with immature myeloid cells in human HNSCC.

### Activation of B7‐H3 in Tgfbr1/Pten 2cKO mouse model

**Figure 3:**
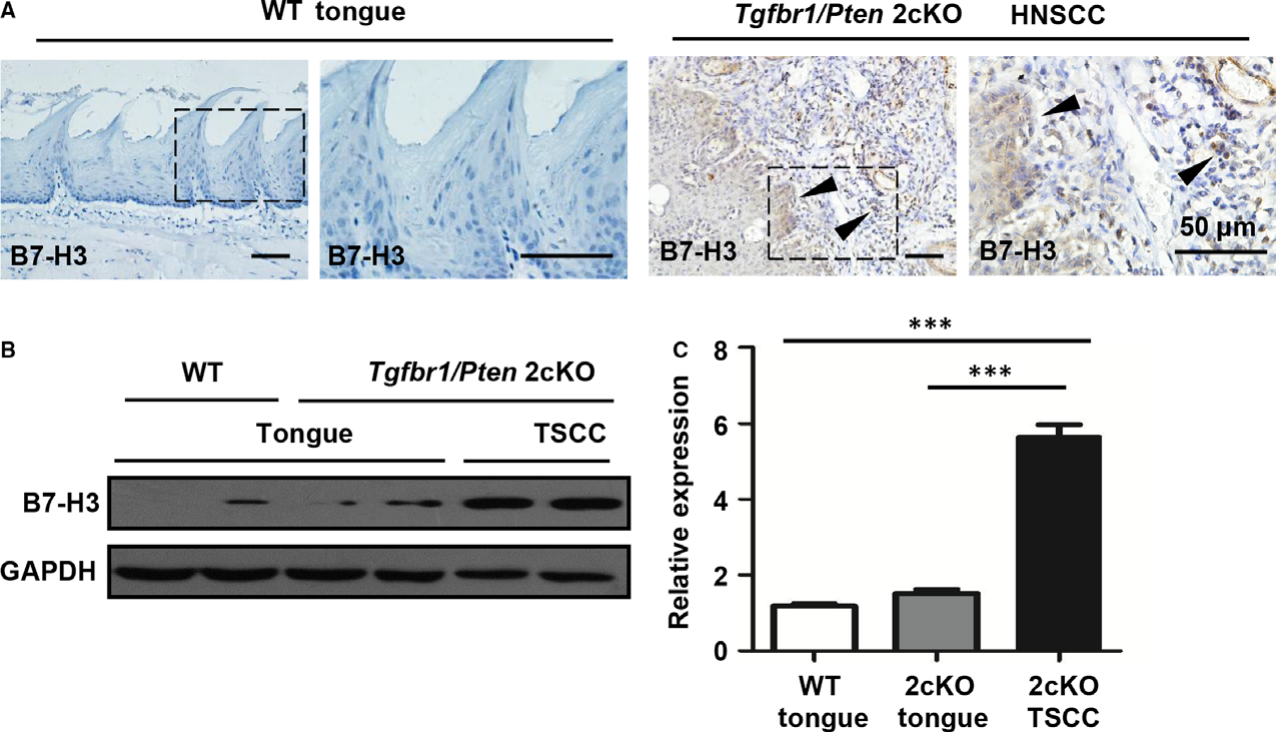
Increased B7‐H3 in *Tgfbr1/Pten* 2cKO mouse HNSCC model. (**A**) Representative immunohistochemical staining of B7‐H3 (arrowhead) in wild‐type (WT) tongue, *Tgfbr1/Pten* 2cKO tongue and 2cKO tongue squamous cell carcinoma (TSCC). Scale bar, 50 μm. (**B**) Western blotting probed with B7‐H3, with GAPDH as a loading control. (**C**) Quantification of B7‐H3 Western blotting relative expression in WT tongue, *Tgfbr1/Pten* 2cKO tongue and 2cKO TSCC (mean ± S.E.M.,* n* = 5 mice, unpaired *t*‐test, ***P < 0.001).

*PTEN* loss has been confirmed as a frequent molecular event in HNSCC 2. Conditional loss of the tumour suppressor *Tgfbr1* in epithelial cells, which abrogates TGF‐β signalling, leads to accumulation of TGF‐β1 ligands in stromal cells 25. A combined deletion of important tumour suppressors *Pten* and *Tgfbr1* (2cKO) leads to a fast and full penetration of HNSCC tumorigenesis in these mice 25. Pathologically, the HNSCC mice were similar to human HNSCC with abundant infiltration of inflammatory cells 25. In tumour‐bearing mice, a significant increase in CD11b ^+^Gr1^+^ and CD11b^+^F4/80^+^ cell populations was found in the tumour and spleen 21. Considering the negative role and rather high expression of B7‐H3 in human HNSCC, we subsequently analysed the expression of B7‐H3 in this mouse model. Indeed, immunostaining of B7‐H3 was significantly increased in 2cKO mouse HNSCC as compared with 2cKO normal oral mucosa as well as wild‐type normal oral mucosa (Fig. 3A). The Western blotting analysis also showed an increased expression consistent with the immunostaining (Fig. 3B and C). Collectively, these results indicate that B7‐H3 expression is activated in *Tgfbr1/Pten* 2cKO mouse model.

### B7‐H3 blockade delayed tumorigenesis in Tgfbr1/Pten 2cKO HNSCC mouse model

**Figure 4:**
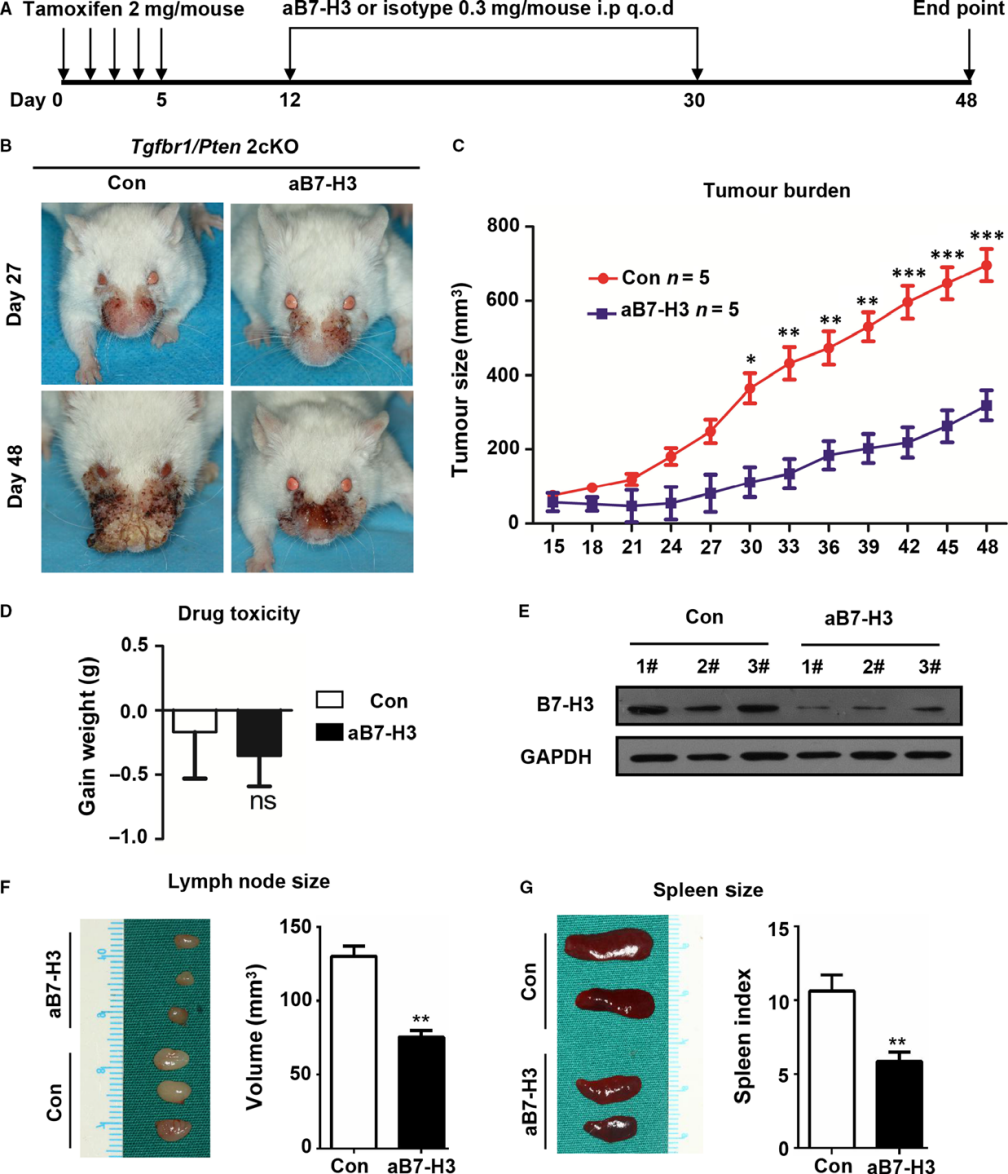
B7‐H3 inhibition attenuates tumour growth in *Tgfbr1/Pten* 2cKO mice. (**A**) Schematic shows chemopreventive experimental therapeutic treatment of *Tgfbr1/Pten* 2cKO mice with B7‐H3 or isotype rat IgG1 (0.3 mg/mouse, i.p.; *n* = 5 mice) every other day. (**B**) Representative photograph of head and neck tumour of the B7‐H3 blockade (aB7‐H3) groups and isotype control groups on day 27 and day 48 after tamoxifen gavage. (**C**) Average volumes of HNSCC on control and aB7‐H3‐treated mice (mean ± S.E.M., unpaired *t*‐test, *P < 0.05, **P < 0.01; ***P < 0.001). (**D**) Quantification of the weight changes in the aB7‐H3 treatment and control groups (*n* = 5 mice, unpaired *t*‐test) (**E**); aB7‐H3 treatment decreased B7‐H3 in *Tgfbr1/Pten* 2cKO mice as compared with the control group as indicated by Western blot with GAPDH as a loading control. (**F**) Decrease in drainage lymph node size and (**G**) reduced spleen index in the aB7‐H3 treatment group as compared with the isotype control group (mean ± S.E.M., unpaired *t‐*test, **P < 0.01).

To test the effect of B7‐H3 on the progression of HNSCC, we performed chemopreventive investigation on the *Tgfbr1/Pten* 2cKO mice. The mice received an intraperitoneal injection of B7‐H3‐blocking antibody (MJ18, 0.3 mg per mouse; *n* = 5 mice) or isotype control rat IgG1 (HRPN, 0.3 mg per mouse; *n* = 5 mice) every other day (Fig. 4A). Tumour growth was assessed every other day after tamoxifen gavage. We observed that blockade of B7‐H3 was sufficient to reduce the tumour burden of head and neck (Fig. 4B and C). Meanwhile, B7‐H3 blockade caused no additional cytotoxicity as compared with the isotype control treatment group (Fig. 4D). And the on‐target effects of B7‐H3 antibody treatment were detected by the Western blotting of tumour tissues (Fig. 4E). Together, these results show that inhibition of B7‐H3 suppresses HNSCC progression. In addition, B7‐H3 blockade effectively rescued immunosuppressive status as indicated by a decreased size of lymph node and reduced spleen index (Fig. 4F and G).

### B7‐H3 blockade reduced MDSCs and M2 macrophages in the Tgfbr1/Pten 2cKO HNSCC mouse model

**Figure 5:**
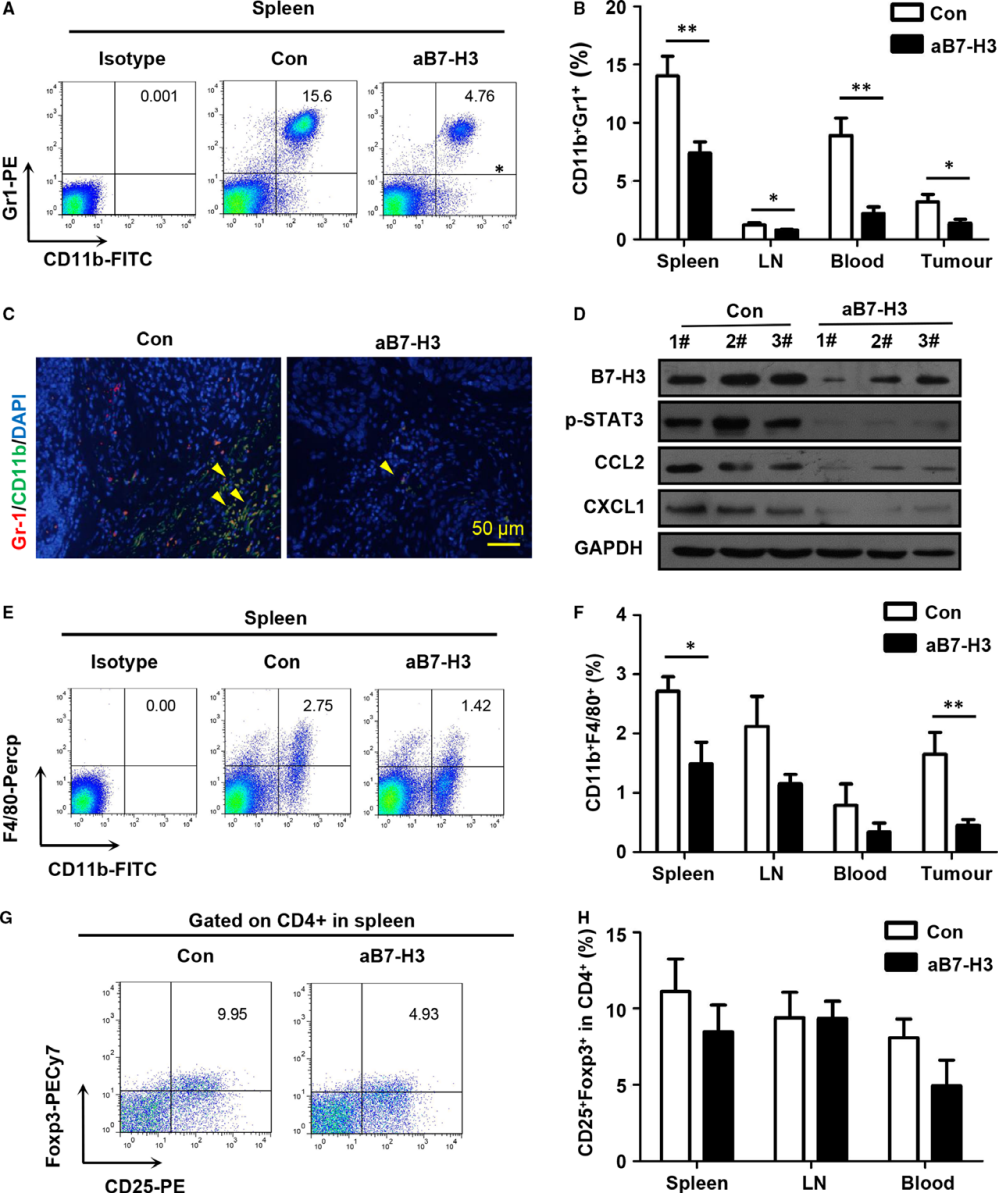
B7‐H3 inhibition reduces MDSCs and TAMs in *Tgfbr1/Pten* 2cKO mice. (**A**) Representative flow cytometry plots of CD11b^+^Gr1^+^ MDSCs in the spleen. (**B**) Quantification of MDSCs from the spleen, lymph node (LN), peripheral blood and tumour on day 48 after tamoxifen gavage (mean ± S.E.M., unpaired *t*‐test, *P < 0.05, **P < 0.01). (**C**) Representative immunofluorescence of CD11b^+^Gr1^+^ MDSCs (yellow arrows) in *Tgfbr1/Pten* 2cKO mouse HNSCC in the aB7‐H3 group and isotype control group. (**D**) Reduced phosphorylation of p‐STAT3^T705^, B7‐H3, CCL2 and CXCL1 in the aB7‐H3 group as compared with the control group with GAPDH as a loading control. (**E**) Representative flow cytometry plots of CD11b^+^F4/80^+^ TAMs in the spleen. (**F**) Quantification of CD11b^+^F4/80^+^ TAMs in the spleen, blood, DLN and tumour (mean ± S.E.M., unpaired *t*‐test, *P < 0.05, **P < 0.01). (**G**) Representative flow cytometry plots of CD4^+^ CD25^+^Foxp3^+^ Tregs in the spleen. (**H**) Quantification of CD4^+^ CD25^+^Foxp3^+^ Tregs in the spleen, LN and blood (mean ± S.E.M., unpaired *t*‐test).

Because we found that B7‐H3 blockade has a significant effect on peripheral immune organs, we suggested that the immunosuppressive cells are potentially involved in the biological process. Considering that MDSCs and M2 macrophages can function as a negative regulator of antitumour immune response in HNSCC, we focused on whether therapeutic blockade of B7‐H3 could decrease MDSCs and M2 macrophages in this mouse model. The single‐cell suspension from the spleen, LN, blood and tumour was stained with antibody for CD11b, Gr‐1 and F4/80. Indeed, B7‐H3 blockade remarkably decreased CD11b^+^Gr1^+^MDSCs in macroenvironment and microenvironment (Fig. 5A and B). And double immunostaining of CD11b and Gr‐1 indicated that B7‐H3 blockade effectively decreased MDSCs in 2cKO mouse HNSCC (Fig. 5C). Western bolt analysis supports that blockade of B7‐H3 could effectively attenuate the recruitment of MDSCs and M2 macrophages by reducing chemokine CXCL1 and CCL2 in the tumour (Fig. 5D). Similarly, CD11b^+^F4/80^+^ M2 macrophages were significantly reduced in the spleen and tumour in the treated group as compared with the control (Fig. 5E and F). In addition, B7‐H3 blockade did not lead to significant regression of CD4^+^CD25^+^Foxp3^+^ Tregs in immune organs and peripheral blood (Fig. 5G and H). Together, these observations suggest that B7‐H3 antibody treatment can effectively reduce MDSCs and M2 macrophages in HNSCC mouse model.

### B7‐H3 blockade improved T cell effector function in Tgfbr1/Pten 2cKO HNSCC mouse model

**Figure 6:**
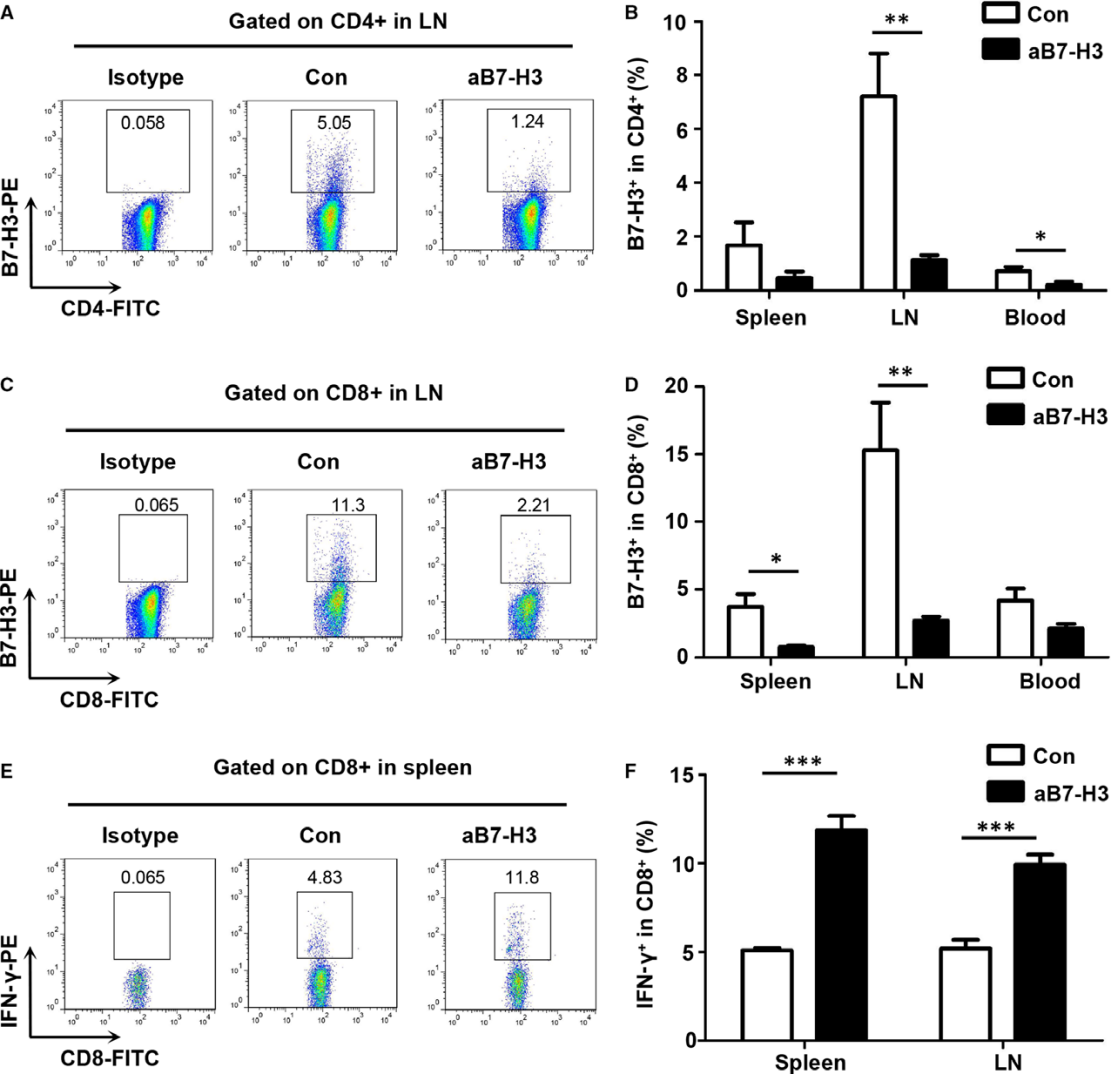
B7‐H3 blockade improved effector T cell function in *Tgfbr1/Pten* 2cKO mouse HNSCC model. (**A**) Representative flow cytometry of B7‐H3^+^ CD4^+^T cells in the lymph node (LN). (**B**) Quantification of the percentage of B7‐H3^+^ CD4^+^T cells in total CD4^+^T cells from the spleen, lymph node (LN) and blood of mice (mean ± S.E.M., unpaired *t*‐test, *P < 0.05, **P < 0.01). (**C**) Representative flow cytometry of B7‐H3^+^ CD8^+^T cells in the lymph node (LN). (**D**) Quantification of the percentage of B7‐H3^+^ CD8^+^T cells in total CD8^+^T cells from the spleen, lymph node (LN) and blood of mice (mean ± S.E.M., unpaired *t*‐test, *P < 0.05, **P < 0.01). (**E**) Representative flow cytometry of IFN‐γ^+^ CD8^+^T cells in the spleen. (**F**) Quantification of the percentage of IFN‐γ^+^ CD8^+^T cells in total CD8^+^T cells from the spleen and lymph node of mice (LN) (mean ± S.E.M., unpaired *t*‐test,***P < 0.001).

To determine whether aB7‐H3 had a broader role in activating T cells, single‐cell suspension from the spleen, LN, blood and tumour was stained with CD4, CD8 and B7‐H3 antibodies. Quantitative flow cytometry results demonstrated that B7‐H3 blockade significantly reduced the B7‐H3^+^CD4^+^ and B7‐H3^+^CD8^+^T cells in the aB7‐H3‐treated mice as compared with the control (Fig. 6A–D). Further, mice receiving aB7‐H3 demonstrated higher levels of IFN‐γ production in response to B7‐H3 blockade as compared with the control group (Fig. 6E and F). The combined results suggest that B7‐H3 blockade significantly enhanced the antitumour effect of T cells.

## Discussion

The immune system plays a key role in the progression of HNSCC as initially suggested by the expansion of immunosuppressive populations (i.e. MDSCs and TAMs) at the tumour site and in the blood 4. Therapeutic manipulation of the immune system and its response, by corollary, may also play a significant role in the treatment of HNSCC 5. In the present study, we describe both analyses of clinical data and preclinical HNSCC mouse model testing the hypothesis that blocking B7‐H3 could perform an antitumour function by increasing IFN‐γ^+^CD8^+^ T cells. Interestingly, we also demonstrated that the inhibition of B7‐H3 could reduce the accumulation of immature myeloid cells, including MDSCs and TAMs.

The rapid‐fire clinical successes from blocking the ligands of B7 superfamily, such as CTLA‐4 and PD‐1, have opened prospects for extending this potential cancer immunotherapy by inhibiting more recently discovered checkpoint ligands and receptors 26, 27, 28. B7‐H3, a newly identified member of the B7 family of molecules, has been linked to head and neck cancer, and its overexpression has been correlated with poor survival 23, 29, 30. Consistent with those reports, our immunohistochemical staining indeed indicates overexpression of B7‐H3 in the HNSCC tissues as compared with oral mucosa or the dysplasia. Of particular interest, high expression of B7‐H3 confers poor overall survival of patients with HNSCC. However, the poor prognosis conferred by B7‐H3 may not depend on lymph node metastasis, poor differentiation or advanced tumour size. Keeping in mind that this is a rather small cohort with 165 primary HNSCC patients with majority of HPV‐ status, a large cohort with more diverse patient population is needed in the future studies.

B7‐H3 blockade may offer a feasible antitumour function by reinvigorating antitumour immune response. B7‐H3 inhibits T cell activation and proliferation and is correlated with clinicopathological features in tumour, which suggested that B7‐H3 may play a role as an adversary in antitumour immunity 31, 32, 33. Restoration of effector CD4 + and CD8^+^ T cells may be suppressed in cancer through the recruitment of MDSCs 34 or TAMs 35. Blocking B7‐H3 with monoclonal Ab (MJ18, rat IgG1) shows a remarkable increase in the accumulation of IFN‐γ^+^CD8^+^ T cells in the spleen and LN. Similar results were also reported indicating that B7‐H3 directly augments CD8^+^ T cell effector function and may play an important role in overcoming CD8^+^ T cell immunoregulation to acquire aggressive growth 36, 37.

Immature myeloid cells, including MDSCs and TAMs, are essential components of the tumour stroma which are deeply intertwined in the course of tumour development and tumour recurrence 38 and have emerged as key immune modulators that suppress antitumour immune responses through a variety of mechanisms 38, 39. Clinical studies have also demonstrated that MDSCs are critical tumour‐promoting immune cells correlating with poor clinical outcome, including tumour metastasis and survival 40, 41, 42, 43. Our previous study revealed that the accumulation of MDSCs and TAMs significantly associated with activation of immune checkpoint molecule PD‐1 44, CTLA‐4 21 and B7‐H4 45. By microarray analysis, a panel of cytokines which were significantly increased in *Tgfbr1/Pten* 2cKO mice are functionally recruiting myeloid cells, such as *Ccl2, Ccl3, Cxcl1* and *Csf1* 16, 44. There are many factors known to be involved in MDSC recruitment, including CXCL1, CXCL5, CCL2, CCL3 and GM‐CSF 46, 47. In our studies, we detected a decrease in MDSCs with the abatement of CCL2 and CXCL1 by inhibiting B7‐H3. This finding coincides with our present results that M2 macrophages were recruited to the site of tumour in our mouse model. Phosphorylated activation of STAT3 is critical of MDSC expansion and maturation blockade. Recent studies have demonstrated that the phosphorylated level of STAT3 at residue Tyr705 was decreased in B7‐H3 knockdown cells 48 and the expression of p‐STAT3 was significantly higher in B7‐H3 high‐expression melanomas compared with B7‐H3 low‐expression melanomas 49. Interestingly, suppressing STAT3 activation can inhibit macrophage differentiation to M2 phenotype 50. Indeed, our study found that blocking B7‐H3 decreased M2 TAMs and p‐STAT3 in our HNSCC mouse model. In addition to enhanced recruitment and expansion of MDSCs, a recent report indicated that B7‐H3 is exclusively expressed on a subset of intratumoral CD14^+^HLA^*−*^DR^*−*/low^ MDSCs in non‐small‐cell lung carcinoma (NSCLC) 39. The local expression of B7‐H3 in MDSCs may suggest the direct function or subpopulation shifting in MDSCs by B7‐H3, which merits further investigations. Enoblituzumab (MGA271) is an Fc‐optimized monoclonal antibody that targets B7‐H3. Enoblituzumab is currently in phase 1 pharmacokinetics and pharmacodynamics clinical testing (MacroGenics, NCT02475213, NCT01391143 and NCT02381314) in refractory cancer with or without addition of ipilimumab or pembrolizumab.

In summary, the present study identified that B7‐H3 may serve as a potential biomarker for HNSCC and its overexpression may confer poor prognosis of patients with HNSCC. Using immune competent HNSCC mouse model, we have directly observed that targeting B7‐H3 decreased immature myeloid cells (MDSCs and TAMs) and promoted T cell activation in tumour microenvironment and macroenvironment. Thus, our study strongly suggests that B7‐H3 may be a potential target for immune therapy for patients with HNSCC.

## Conflict of interest

The authors declare no conflict of interest.

## Supporting information


**Figure S1.** CD276 gene increased in human HNSCC.
