# Supplementary figures and images for: Selective blockade of B7‐H3 enhances antitumour immune activity by reducing immature myeloid cells in head and neck squamous cell carcinoma
Source: J Cell Mol Med. 2017 Apr 11;21(9):2199–210. doi: 10.1111/jcmm.13143 (PMC5571514; doi:10.1111/jcmm.13143)

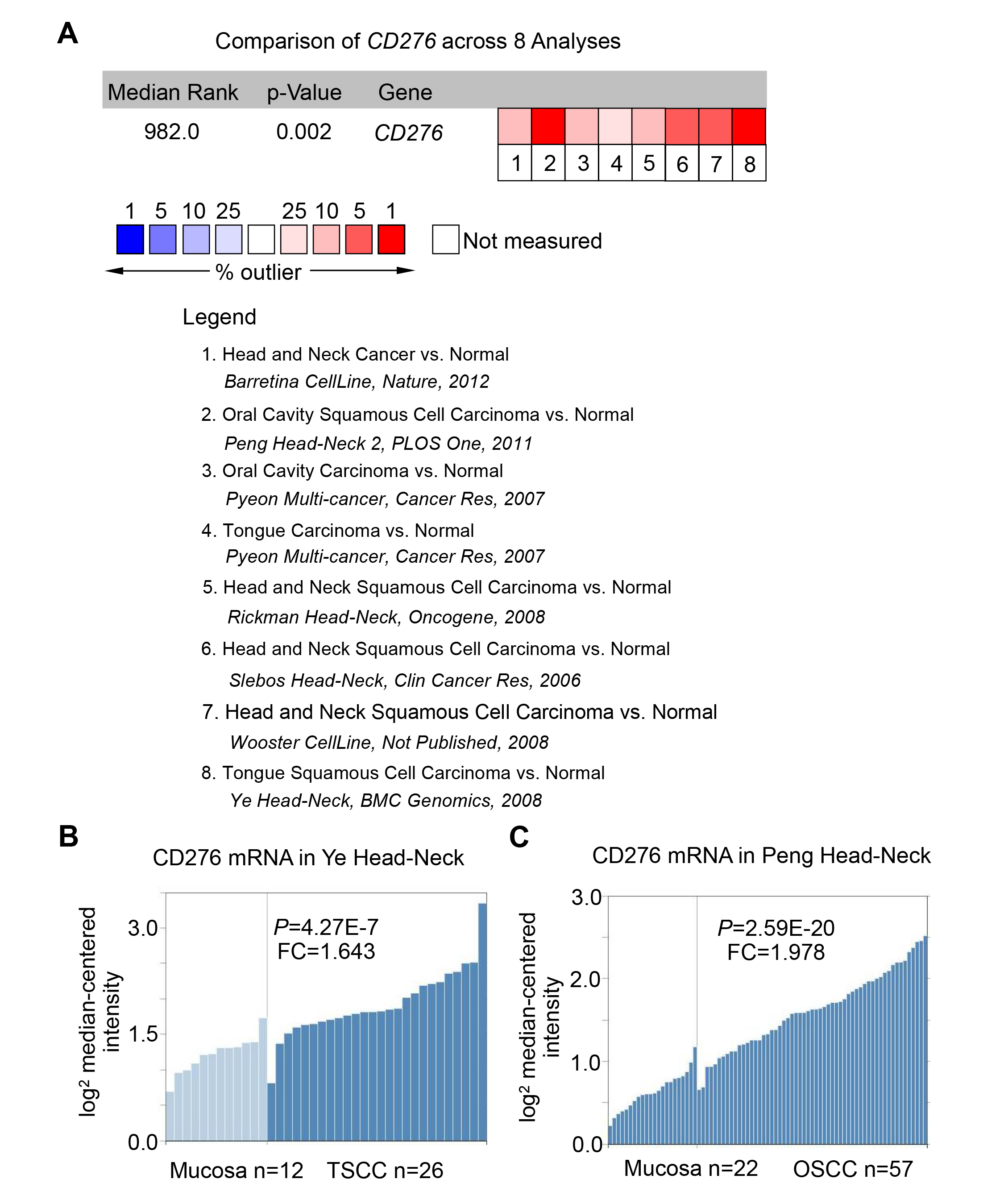

Supplement: Supplementary file 1 — Figure S1. CD276 gene increased in human HNSCC. [file JCMM-21-2199-s001.tif]
